# Chrono-Aerobic Exercise Optimizes Metabolic State in DB/DB Mice through CLOCK–Mitophagy–Apoptosis

**DOI:** 10.3390/ijms23169308

**Published:** 2022-08-18

**Authors:** Zhe Zhang, Xi Li, Jun Zhang, Jing Du, Qiang Zhang, Zhe Ge, Shuzhe Ding

**Affiliations:** 1 Key Laboratory of Adolescent Health Assessment and Exercise Intervention, Ministry of Education, East China Normal University, Shanghai 200241, China; 2 College of Physical Education and Health, East China Normal University, Shanghai 200241, China

**Keywords:** chrono-exercise, molecular clock, mitochondrial quality, apoptosis

## Abstract

Although the benefits of aerobic exercise on obesity and type 2 diabetes are well-documented, the pathogenesis of type 2 diabetes and the intervention mechanism of exercise remain ambiguous. The correlation between mitochondrial quality and metabolic diseases has been identified. Disruption of the central or peripheral molecular clock can also induce chronic metabolic diseases. In addition, the interactive effects of the molecular clock and mitochondrial quality have attracted extensive attention in recent years. Exercise and a high-fat diet have been considered external factors that may change the molecular clock and metabolic state. Therefore, we utilized a DB/DB (BSK.Cg-Dock7m +/+ Leprdb/JNju) mouse model to explore the effect of chrono-aerobic exercise on the metabolic state of type 2 diabetic mice and the effect of timing exercise as an external rhythm cue on liver molecular clock-mitochondrial quality. We found that two differently timed exercises reduced the blood glucose and serum cholesterol levels in DB/DB mice, and compared with night exercise (8:00 p.m., the active period of mice), morning exercise (8:00 a.m., the sleeping period of mice) significantly improved the insulin sensitivity in DB/DB mice. In contrast, type 2 diabetes mellitus (T2DM) increased the expression of CLOCK and impaired the mitochondrial quality (mitochondrial networks, OPA1, Fis1, and mitophagy), as well as induced apoptosis. Both morning and night exercise ameliorated impaired mitochondrial quality and apoptosis induced by diabetes. However, compared with morning exercise, night exercise not only decreased the protein expression of CLOCK but also decreased excessive apoptosis. In addition, the expression of CLOCK was negatively correlated with the expression of OPA1 and Fis1. In summary, our research suggests that morning exercise is more beneficial for increasing insulin sensitivity and promoting glucose transport in T2DM, whereas night exercise may improve lipid infiltration and mitochondrial abnormalities through CLOCK–mitophagy–apoptosis in the liver, thereby downregulating glucose and lipid disorders. In addition, CLOCK-OPA1/Fis1–mitophagy might be novel targets for T2DM treatment.

## 1. Introduction

Circadian rhythms provide a selective advantage by anticipating organismal nutrient needs and guaranteeing an optimal metabolic capacity during active hours. The circadian clock is present in most organisms, including mammals. In mammals, the circadian clock orchestrates 24-h oscillations in many physiological processes [1]. These rhythms are driven by light information sensed by the eyes and processed by the central clock located in the suprachiasmatic nucleus (SCN). However, peripheral tissues such as the liver and skeletal muscles have their own clocks and exhibit independent circadian oscillations, synchronized to the central clock through nervous and hormonal signals [2]. Disturbing circadian rhythms at the peripheral organ or whole-body level increases the risk of developing chronic metabolic diseases, such as type 2 diabetes mellitus (T2DM). Furthermore, in peripheral tissues, including the liver and skeletal muscle, molecular clocks can be entrained by cues such as light, feeding time, high-fat diet, and activity [3,4,5,6]. Scheduled bouts of exercise result in a significant shift in clock gene expression in the peripheral tissues [7]. The potential impact of physical activity might be an entrainment cue for peripheral tissues and also suggests that entrainment cues for peripheral tissues may be therapeutic strategies for abnormal lipid metabolism in circadian disruption [8].

The circadian clock regulates many transcriptional–translational processes that influence whole cell metabolism, particularly the mitochondrial quality [9]. To adapt to an ever-changing environment, mitochondria are highly dynamic in form and function, and the loss of this flexibility is liable to result in metabolic diseases. Recent studies have indicated that changes in the mitochondrial dynamics (fusion and fission) and morphology are dependent on a viable circadian clock [2]. Neufeld-Cohen et al. [10] found that nearly 38% of 590 mitochondrial proteins, including mitochondrial dynamics, are regulated by the circadian clock in the liver.

Based on these findings, we hypothesized that chrono-exercise might be an important cue for entraining circadian rhythms in T2DM livers and may be a useful therapeutic strategy for T2DM by ameliorating molecular clock disruption and aberrant mitochondrial quality. DB/DB (BSK.Cg-Dock7m +/+ Leprdb/JNju) mice are a genetic model of type 2 diabetes and obesity, because they lack a functional leptin receptor. In this study, DB/DB mice were forced to run at 8:00 a.m. and 8:00 p.m. for 8 weeks. We found that exercise reduced the body weight, blood glucose, and serum cholesterol levels of T2DM mice, and compared with night exercise (8:00 p.m.), morning exercise (8:00 a.m.) significantly improved the insulin sensitivity and glucose transport of T2DM mice. Type II diabetes increases the protein level of liver rhythm molecule CLOCK and impairs the mitochondrial morphology, dynamics (OPA1 and Fis1), and mitophagy, which can be optimized by two differently timed exercises. However, compared with morning exercise, night exercise decreased the protein expression of CLOCK and excessive apoptosis. In addition, the protein level of CLOCK was negatively correlated with the protein levels of Fis1 and OPA1. Hence, morning exercise is more advantageous in increasing insulin sensitivity in type II diabetic mice, whereas night exercise may improve liver fat infiltration and mitochondrial abnormalities through CLOCK–mitophagy–apoptosis, thereby reducing the blood glucose and lipid levels. Meanwhile, CLOCK–OPA1/Fis1–mitophagy might be promising targets for T2DM treatment.

## 2. Results

Morning exercise improves the insulin sensitivity in diabetic mice.

**Figure 1:**
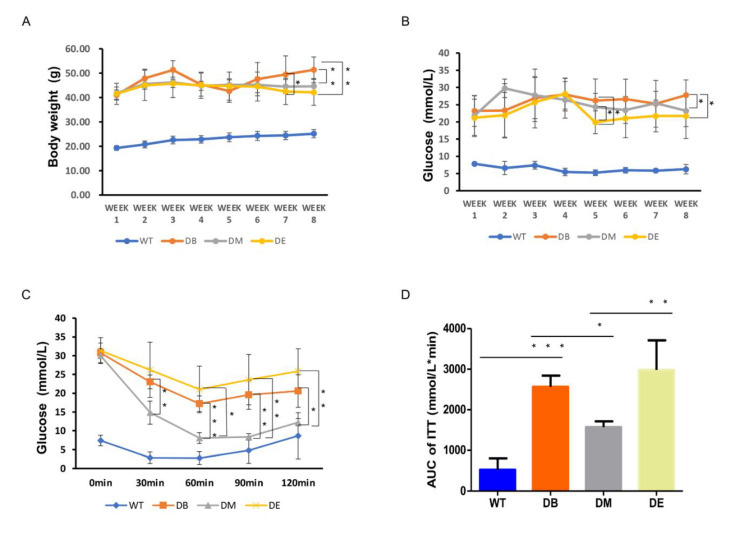

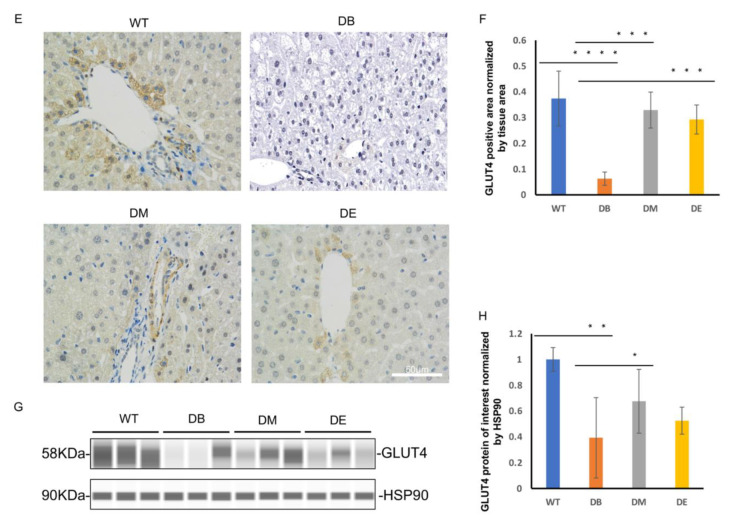
Chrono-exercise ameliorates blood glucose, and morning exercise improves insulin sensitivity in diabetic mice. (**A**) Body weight in WT, DB, DM, and DE mice. * *p* < 0.05 and ** *p* < 0.01, *n* = 8–12. (**B**) Changes in fasting blood glucose during the test. * *p* < 0.05, *n* = 8–10. (**C**) Blood glucose levels in the ITT test of groups WT, DB, DM, and DE mice after running. * *p* < 0.05, ** *p* < 0.01, and *** *p* < 0.001, *n* = 3–4. (**D**) Area under the curve of the ITT graph of (**C**). * *p* < 0.05, ** *p* < 0.01, and *** *p* < 0.001, *n* = 4. (**E**) GLUT4 was compared between different groups by GLUT4 antibody staining, *n* = 3. (**F**) Statistical evaluation of GLUT4 staining of (**E**), *** *p* < 0.001 and **** *p* < 0.0001. (**G**) Wes^TM^ of GLUT4 expression in liver from groups WT, DB, DM, and DE mice. (**H**) Statistical evaluation of GLUT4 expression in the liver. * *p* < 0.05 and ** *p* < 0.01, *n* = 3.

The body weight of the diabetic mice remained higher than that of the WT mice during the experiment (Figure 1A). Although the body weights of the DM and DB groups also remained high during the experiment, compared with the DB group, we found that the body weight of DM mice decreased on exercise weeks 7 (*p* < 0.05) and 8 (*p* < 0.01), and the body weight of DE mice only decreased on exercise week 8 (*p* < 0.01), with no difference observed between DM and DE. These results suggest that both morning and night exercise are beneficial for weight loss in diabetic mice.

The blood glucose level of diabetic mice was much higher than that of WT mice. At exercise week 5, the blood glucose levels in the DE group were significantly lower than those in the DB and DM groups (*p* < 0.05; Figure 1B). At exercise week 8, the blood glucose in the DM and DE groups also significantly decreased compared with that in the DB group (*p* < 0.05), but no differences were observed between the DM and DE groups (Figure 1B).

Insulin sensitivity was assessed in diabetic and WT mice using ITT. The blood glucose levels of diabetic mice were significantly higher in the DB, DM, and DE groups than in WT mice throughout the test (Figure 1C). The blood glucose level was significantly lower in the DM group than in the DB group 30 min after insulin injection until the end of the ITT test (*p* < 0.01, *p* < 0.001, *p* < 0.01, and *p* < 0.05, respectively; Figure 1C). The blood glucose level of DM mice was significantly lower than that of DE mice 60 min after injecting insulin until the end of the ITT test (*p* < 0.05, *p* < 0.01, and *p* < 0.01, respectively; Figure 1C). The area under the curve (AUC) of the ITT graph was significantly higher in the DB group than in the WT group (*p* < 0.001; Figure 1D). Moreover, the AUC of the ITT graph was significantly lower in the DM group than in the DB (*p* < 0.05; Figure 1D) and DE groups (*p* < 0.01; Figure 1D). Therefore, both morning and night exercises reduced the blood glucose levels in diabetic mice, but only morning exercise increased the insulin sensitivity in diabetic mice.

Since we found that the blood glucose levels of DM and DE mice were significantly lower than those of DB mice after exercise intervention, and increased insulin sensitivity was also found in DM mice, we hypothesized that exercise may improve glucose transporter 4 (GLUT4) expression to decrease blood glucose and increase insulin sensitivity. We used immunohistochemistry and Wes^TM^ to detect GLUT4 and quantified the staining area and the expression of GLUT4 in the liver. Compared with WT mice, the GLUT4 signal (yellow or brown) almost disappeared in the DB mice (*p* < 0.0001; Figure 1E,F). Compared with DB, a greater GLUT4 signal was observed in the DM and DE groups (*p* < 0.001; Figure 1E,F). Similarly, the Wes^TM^ results also showed that, compared with WT mice, the expression of GLUT4 significantly decreased in DB mice (*p* < 0.01; Figure 1G,H). Compared with DB mice, the expression of Glut4 increased in DM mice (*p* < 0.05; Figure 1G,H). Although there was no difference between the DB and DE groups, compared with the DB group, the protein level of GLUT4 in the DE group still increased (Figure 1G,H), which was consistent with the immunohistochemical results of GLUT4 (Figure 1E,F). The above results suggest that morning exercise is more beneficial for promoting glucose transport in T2DM, which is consistent with insulin sensitivity.

### Exercise Downregulates Serum T-CHO in Diabetic Mice

**Figure 2:**
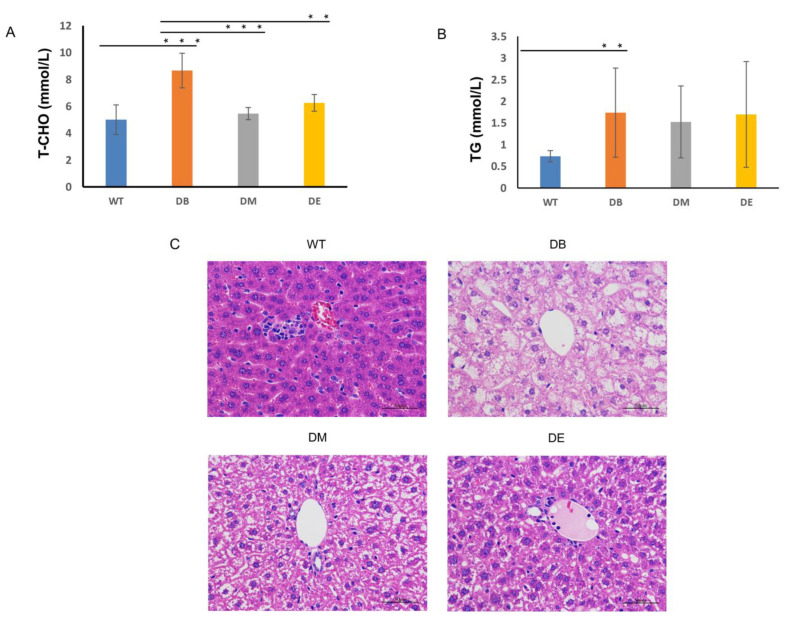
Exercise decreases diabetes-induced hypercholesterolemia. (**A**) T-CHO levels in serum from WT, DB, DM, and DE mice. ** *p* < 0.01 and *** *p* < 0.001, *n* = 6. (**B**) TG levels in serum from WT, DB, DM, and DE mice. ** *p* < 0.01, *n* = 6. (**C**) HE staining images of livers from WT, DB, DM, and DE mice (*n* = 3).

We found that both morning (DM) and night (DE) exercise significantly decreased the blood glucose levels. We wondered whether chrono-exercise also differentially affected the serum lipid levels. The serum lipid, T-CHO, and TG levels were assessed using Nanjing Jiancheng kits. We found that the T-CHO level of the DB group was significantly increased compared with that of the WT group (*p* < 0.001; Figure 2A). The T-CHO levels of both the DM (*p* < 0.001) and DE groups (*p* < 0.01) were significantly decreased compared to that of the DB group (Figure 2A). Although the TG level of the DB group was significantly higher than that of the WT group (*p* < 0.01), there was no difference between the DM and DE groups compared to the DB group (Figure 2B).

We also applied HE staining to observe the liver structure and fat cavitation in diabetic mice. Large amounts of fat infiltrate were observed in the DB, and the liver structure was severely damaged (Figure 2C). Morning and night exercise ameliorated fat infiltration and serious structural damage, and the effect of night exercise was better than that of morning exercise (Figure 2C).

Night exercise is better than morning exercise in relieving the change in the CLOCK protein level caused by diabetes.

**Figure 3:**
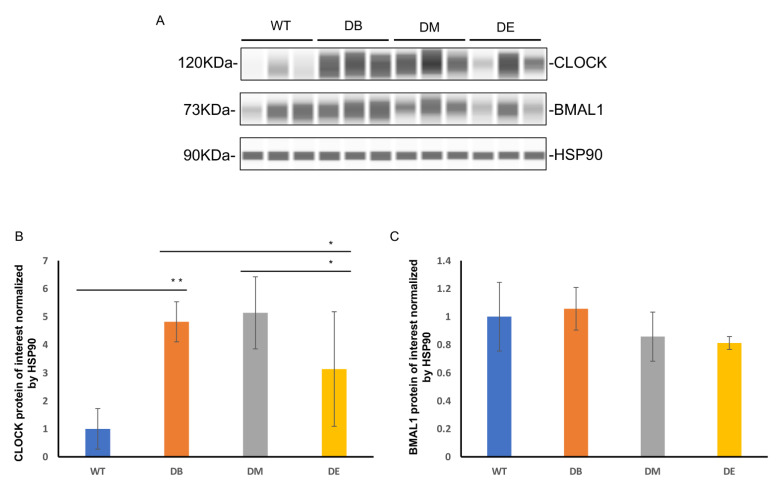
Night exercise is better than morning exercise in relieving the change of CLOCK expression caused by diabetes. (**A**) Wes^TM^ of CLOCK and BMAL1 expression in livers from groups WT, DB, DM, and DE mice. (**B**,**C**) Statistical evaluation of CLOCK and BMAL1 expression in the liver, as shown in * *p* < 0.05 and ** *p* < 0.01, *n* = 3–4.

As mentioned previously, disturbed circadian rhythms in organs or the whole body are associated with abnormal metabolic states, such as high-fat diet, obesity, and T2DM. Scheduled bouts of exercise result in a significant shift in clock gene expression in the peripheral tissues [7]. The liver is the main regulatory organ that participates in the synthesis and decomposition of cholesterol; therefore, serum cholesterol levels also reflect liver lipid metabolism. The liver is one of the most important metabolic organs; it has its own clock and exhibits independent circadian oscillations [2]. Therefore, we speculated that the effect of rhythmic exercise may be observed in the liver. We measured the expression of the central molecules involved in the molecular clock—CLOCK and BMAL1—using Wes^TM^ automatic protein expression analysis (Figure 3A–C). Interestingly, we found that diabetes and exercise are differentially associated with these two molecules. Diabetes increased the expression of CLOCK (*p* < 0.01), but no difference was observed in that of BMAL1 (Figure 3A–C). Compared to the DB group, the expression of CLOCK in the DE group decreased (*p* < 0.05; Figure 3A,B); compared to the DM group, the expression of CLOCK also decreased in the DE group (*p* < 0.05; Figure 3A,B). In addition, no differences in the expression of Bmal1 were observed between the DB, DM, and DE groups (Figure 3A–C).

Exercise ameliorates abnormal mitochondrial networks and morphology.

**Figure 4:**
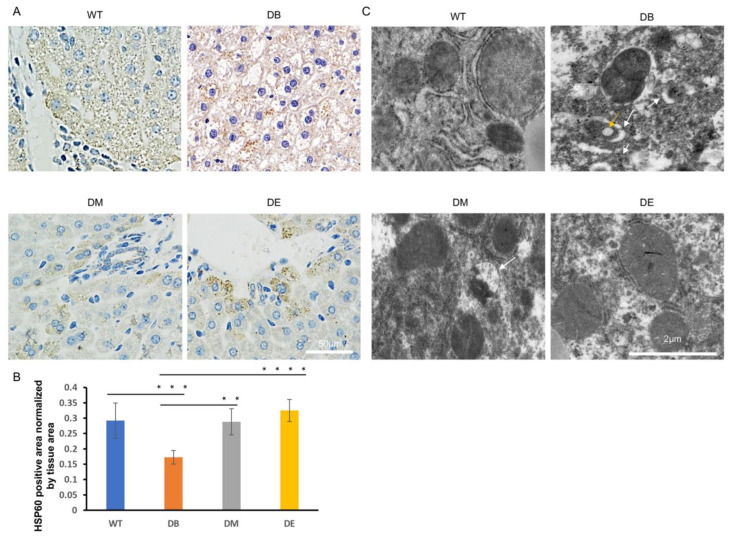
Night exercise is better than morning exercise in ameliorating abnormal mitochondrial networks and morphology caused by diabetes. (**A**) HSP60 staining images of livers from WT, DB, DM, and DE mice (*n* = 3). (**B**) Statistical evaluation of HSP60 staining of (**A**), ** *p* < 0.01, *** *p* < 0.001 and **** *p* < 0.0001. (**C**) Electron microscopy images of livers from WT, DB, DM, and DE mice (*n* = 3). The yellow arrow indicates the mitochondrial lipid content. The white arrows indicate the outer compartment swollen mitochondria.

The mitochondrial networks and morphology in the liver were observed using HSP60 immunostaining and electron microscopy (EM). HSP60 is an important marker of the mitochondrial matrix and is normally used to indicate mitochondrial networks and the quantity. In this study, we found that the mitochondrial networks were significantly destroyed and nonconsecutive in the DB group (Figure 4A). The HSP60-positive area per unit area of tissue in the DB group was significantly lower than that in the WT group (*p* < 0.001; Figure 4A,B). After exercise intervention, the mitochondrial networks were significantly improved (Figure 4A,B). The HSP60-positive area was also significant higher in the DM (*p* < 0.01; Figure 4A,B) and DE groups (*p* < 0.0001; Figure 4A,B) than in the DB group, indicating that exercise increased the mitochondrial networks in diabetic mice. The morphology of individual mitochondria was observed using an electronic microscope. The images suggested that the diabetes-induced mitochondrial lipid content (indicated by the yellow arrow in Figure 4C) and outer compartment swelling (indicated by the white arrow in Figure 4C) were ameliorated after exercise intervention (Figure 4C). However, consistent with the HE staining results, more abnormal mitochondria were found in DM than in DE mice, suggesting that the effect of night exercise on improving the mitochondrial morphology is better than that of morning exercise. These results indicate that both morning and night exercise ameliorate liver structure defects and abnormal mitochondrial networks and morphology, but night exercise has advantages over morning exercise in improving the diabetic liver structure and mitochondrial morphology.

Diabetes dramatically impairs the mitochondrial quality, and exercise ameliorates excessive mitophagy caused by diabetes.

**Figure 5:**
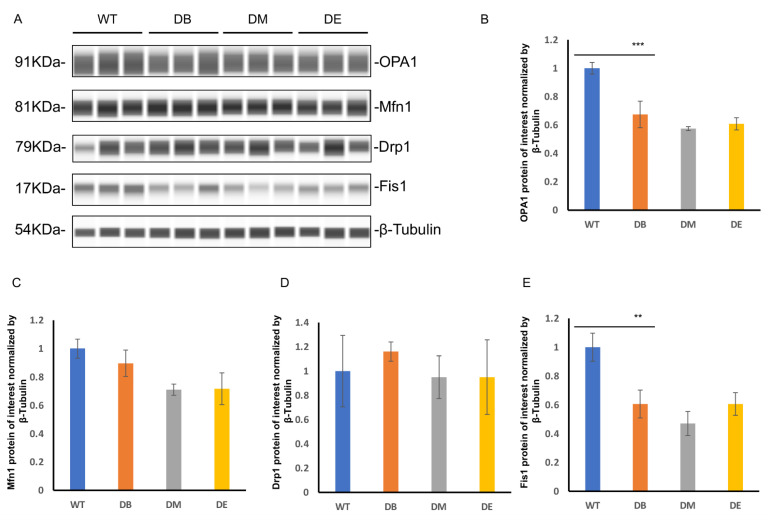

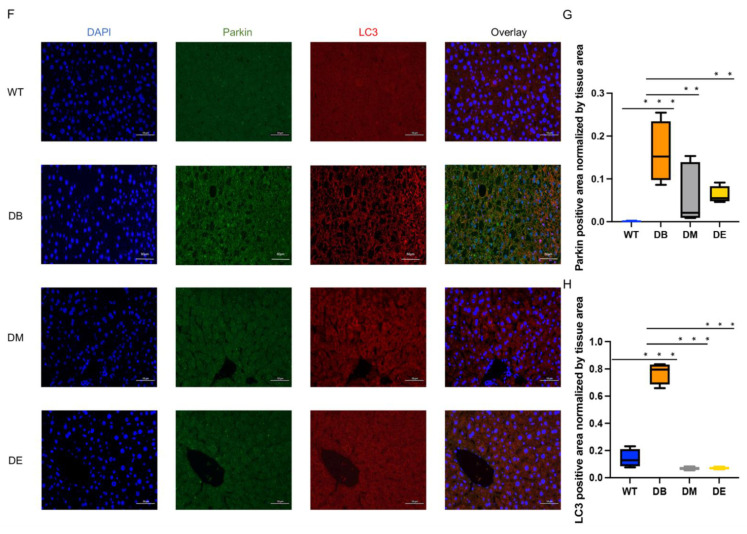
Diabetes dramatically impairs mitochondrial quality, and exercise ameliorates excessive mitophagy caused by diabetes. (**A**) Wes^TM^ of OPA1, Mfn1, Drp1, and Fis1 expression in livers from WT, DB, DM, and DE mice. (**B**) Statistical evaluation of OPA1 expression in livers. *** *p* < 0.001, *n* = 3. (**C**) Statistical evaluation of Mfn1 expression in livers, *n* = 3 to 4. (**D**) Statistical evaluation of Drp1 expression in livers, *n* = 3. (**E**) Statistical evaluation of Fis1 expression in livers. ** *p* < 0.01, *n* = 3. (**F**) Representative images of Parkin and LC3 in livers from WT, DB, DM, and DE mice (*n* = 3). (**G**) Statistical evaluation of Parkin of (**F**), ** *p* < 0.01 and *** *p* < 0.001. (**H**) Statistical evaluation of LC3 of (**F**), *** *p* < 0.001. Scale bar: 50 μm.

The mitochondrial dynamics include fusion and fission. Since we found obvious abnormal mitochondrial morphology, we further assessed the expression of mitochondrial fusion molecules (OPA1 and Mfn1) and mitochondrial fission molecules (Drp1 and Fis1) by measuring liver lysates prepared from WT, DB, DM, and DE mice using the Wes^TM^ automatic protein expression analysis (Figure 5A–E). These analyses revealed that diabetes significantly reduced the expression of OPA1 (*p* < 0.001) and Fis1 (*p* < 0.01) (Figure 5A,B,E) in the liver, suggesting that diabetes impaired mitochondrial dynamics, which is consistent with the abnormal mitochondrial networks and morphology observed in diabetic mice (Figure 4A–C). However, there were no changes in the expression of Mfn1 and Drp1 in either group (Figure 5A,C,D). Compared to the DB group, there were no differences in the expression of mitochondrial dynamic molecules of the DM and DE groups (Figure 5A–E).

Mitochondrial dynamics are closely associated with mitophagy. Therefore, we assessed mitophagy through the immunofluorescence colocalization of Parkin and LC3. Unexpectedly, diabetes resulted in a dramatic increase in the Parkin (*p* < 0.001; Figure 5F,G) and LC3-positive area (*p* < 0.001; Figure 5F,H) in the liver. Moreover, morning exercise significantly decreased the Parkin (*p* < 0.01; Figure 5F,G) and LC3 (*p* < 0.001; Figure 5F,H)-positive area. Night exercise also significantly decreased the Parkin (*p* < 0.01; Figure 5F,G) and LC3 (*p* < 0.001; Figure 5F,H)-positive area and was almost the same as that of WT mice. There was no difference between the DM and DE groups (Figure 5F–H). To ensure that the immunofluorescence staining showed a positive signal (Figure 5F), the sections prepared from WT, DB, DM, and DE mice were applied for negative controls. The results showed that almost no signal was observed in the negative controls (Figure S1), indicating that the Parkin and LC3 signals in Figure 5 are credible. These results indicate that exercise relieves excessive mitophagy caused by diabetes.

The changes in the CLOCK protein level are negatively correlated with the changes in the Fis1 and OPA1 protein levels.

**Figure 6:**
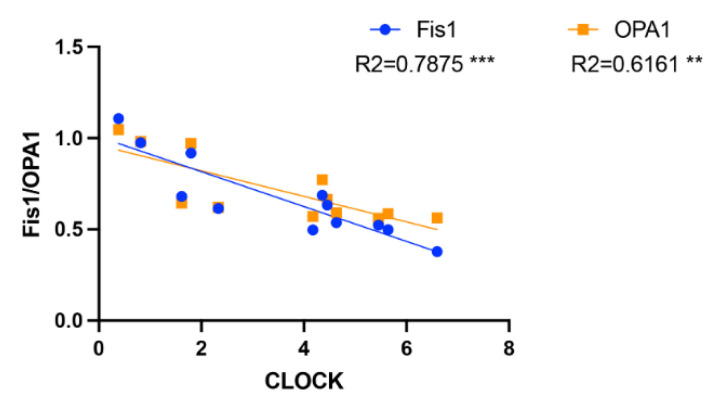
The CLOCK expression is negatively correlated with Fis1 and OPA1 expression. ** *p* < 0.01 and *** *p* < 0.001.

Recent studies have indicated that changes in mitochondrial dynamics (fusion and fission) and morphology are dependent on a viable circadian clock [2]. Neufeld-Cohen et al. [10] found that nearly 38% of 590 mitochondrial proteins, including mitochondrial dynamics, are regulated by the circadian clock in the liver. Here, we found that the changes in the CLOCK protein level were negatively correlated with the changes in the Fis1 (R^2^ = 0.7875, *p* < 0.001) and OPA1 (R^2^ = 0.6161, *p* < 0.01) protein levels, respectively (Figure 6).

Diabetes induces apoptosis, while night exercise is better than morning exercise in relieving apoptosis.

**Figure 7:**
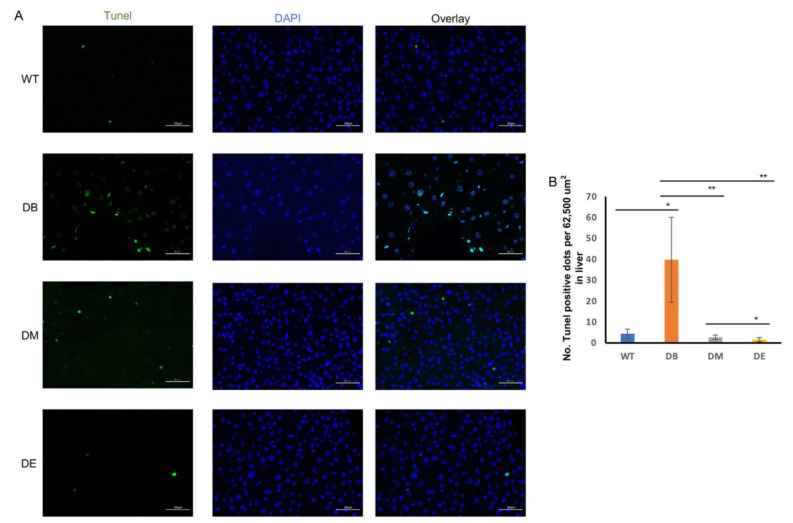
Night exercise is better than morning exercise in relieving apoptosis caused by diabetes. (**A**) Representative images of Tunel in livers from WT, DB, DM, and DE mice. (**B**) Statistical evaluation of Tunel signal in livers from WT, DB, DM, and DE mice. * *p* < 0.05 and ** *p* < 0.01, *n* = 3. Scale bar: 50 μm.

Apoptosis is a common phenomenon when an organ or the whole body is under pathological conditions. Tissue damage can also induce apoptosis. Therefore, we investigated whether diabetes induced apoptosis and whether chrono-exercise improved the pathological state. Liver tissue was prepared for terminal deoxynucleotidyl transferase-mediated nick-end labeling (TUNEL) after the exercise trials. Consistent with the results of LC3–Parkin colocalization, we found that diabetes resulted in a large-scale positive TUNEL signal in the liver (*p* < 0.05; Figure 7A,B). Both morning and night exercises significantly decreased the TUNEL puncta (*p* < 0.01; Figure 7A,B), indicating that exercise relieved diabetes-induced apoptosis. Moreover, consistent with the expression of CLOCK, night exercise had an advantage over morning exercise in relieving apoptosis caused by diabetes (*p* < 0.05; Figure 7A,B).

## 3. Discussion

The regular cycle of changes in body activity exhibited by organisms is called biorhythm, also known as the circadian rhythm. Light, a high-fat diet, and physical activity may serve as entrainment for tissue-specific circadian rhythms. Disturbing circadian rhythms at the organ or whole-body level increases the risk of developing chronic metabolic diseases, such as obesity and T2DM. In contrast, biological circadian rhythm molecules regulate mitochondrial quality through a variety of mechanisms [2,9]. Thus, we hypothesized that different timing exercises may have varying effects on improving the diabetic phenotype through the molecular clock. The mice were forced to run at 8:00 a.m. and 8:00 p.m., which are the sleeping and active periods of mice, respectively. Here, we found that morning exercise is more advantageous than night exercise in improving insulin sensitivity, while night exercise may ameliorate glucose and lipid metabolism defects through CLOCK–mitophagy–apoptosis.

It is well-known that physical exercise is an effective intervention for treating metabolic diseases [11,12,13] such as obesity, hypoglycemia, and diabetes. Although the effects of exercise on body weight and blood glucose were not very significant in this study, the results showed that running still decreased body weight and blood glucose in diabetic mice (Figure 1A,B), which was similar to the results of previous studies [14,15,16]. Prolonged exercise, such as a 12-week exercise intervention, may have more significant effect on weight loss and blood glucose. In addition, it is necessary to explore the effect of exercise in accordance with the circadian rhythm to improve the diabetic phenotype. In our study, the ITT results suggested that morning exercise significantly increased insulin sensitivity, whereas night exercise did not improve insulin sensitivity in diabetic mice (Figure 1C,D), indicating that morning exercise was better than evening exercise in ameliorating insulin resistance. GLUT4 is the principal glucose transporter protein that mediates glucose uptake and plays a key role in the regulation of whole-body glucose homeostasis. The expression or activity of GLUT4 decreases the glucose uptake and utilization, which is an important molecular basis for insulin resistance. Consistent with the ITT results, GLUT4 staining and GLUT4 expression also showed that diabetes significantly reduced the GLUT4-positive area (*p* < 0.0001) and GLUT4 expression (*p* < 0.01) in the liver, which were significantly ameliorated by exercise (Figure 1E–H). Although Glut4 staining and GLUT4 expression showed that there was no difference between the DM and DE groups, morning exercise improved GLUT4 expression in diabetic mice (*p* < 0.05; Figure 1E–H), whereas night exercise did not improve GLUT4 expression in diabetic mice, indicating that morning exercise may also be better than evening exercise in promoting glucose transport in diabetic mice.

Except for blood glucose, glucose and lipid metabolism disorders are typical symptoms in most type 2 diabetes patients. Here, we found that diabetes increased the serum T-CHO and TG levels (Figure 2A,B), suggesting that diabetes impairs lipid metabolism. Although exercise significantly reduced serum cholesterol levels, no difference was found in the effects of morning and night exercise.

The molecular clock exists in almost all cells of an organism and exhibits tissue-specific rhythmic characteristics. The expression and function of the molecular clock in the liver is conducive to maintaining normal lipid metabolism. In contrast, the liver-specific knockout of key rhythmic molecules could induce abnormal lipid metabolism [17], promote diet-induced obesity, and increase the blood glucose levels, which, in turn, induces diabetes [18]. BMAL1 and CLOCK are core molecules of the molecular clock. They form a heterodimer that binds to the E-box regulatory sequence PER/CRY and other genes. Over the past decades, it has become apparent that a functional clock is crucial for maintaining normal glucose homeostasis [19]. The whole-body loss of clock function (e.g., CLOCK or BMAL1 knockout animals) leads to hyperglycemia, glucose intolerance, and, ultimately, obesity and metabolic syndrome [19], whereas ablation of the liver CLOCK impairs glycogenesis and reduces hepatic glucose production [20], indicating the varying consequences of CLOCK dysfunction are tissue-specific [21]. Similarly, our results showed that diabetes significantly increased the expression of CLOCK in liver (*p* < 0.01; Figure 3A,B). In contrast, diabetes did not change the expression of another rhythmic molecule—BMAL1 (Figure 3A,C). The recent characterization of the CLOCK-BMAL1 cistrome revealed that, although CLOCK–BMAL1 binds synchronously to all of its target genes, its transcriptional output is highly heterogeneous [22]. Upon DNA binding during the active phase, CLOCK–BMAL1 promotes chromatin modification by recruiting histone-modifying enzymes to core clock gene promoters and enhancers [23]. We speculated that the varying effect of diabetes on CLOCK and BMAL1 was due to the heterogeneity of histone modifications.

Scheduled bouts of exercise result in a significant shift in clock gene expression in the peripheral tissues [7]. In our study, night exercise not only improved impaired CLOCK expression caused by diabetes (*p* < 0.05) but also decreased CLOCK expression (*p* < 0.05) compared with morning exercise (Figure 3A,B), indicating that night exercise is better than morning exercise in alleviating impaired CLOCK expression induced by T2DM, which is speculated that night exercise is more consistent with the biorhythm of mice. Similar to the effects of diabetes on BMAL1 expression, neither morning exercise or night exercise altered the BMAL1 expression (Figure 3A,C). Mice were forced to run on a treadmill for 8 weeks in this study. Longer training cycles may be necessary to significantly improve the core molecules of the molecular clock [24]. In addition, diabetes and exercise may selectively or characteristically affect the heterodimer of CLOCK and BMAL1.

Damaged liver structure and abnormal mitochondrial dynamics were observed in this study (Figure 2C, Figure 4 and Figure 5). Diabetes induced the obvious lipid content (Figure 2C) and swollen outer compartment in the mitochondria (Figure 4C) and destroyed the mitochondrial networks (Figure 4A,B), fusion, and fission (Figure 5A,B,E), suggesting that diabetes induces lipid infiltration and impairs the mitochondrial morphology and dynamics. Similar to our observations, high-fat diet (HFD) mice also show impaired mitochondrial dynamics in the brain [25]. Interestingly, the increased expression of Fis1 was found in the brains of HFD mice, with no significant difference in OPA1 [25]. However, we found that diabetes reduced the expression of OPA1 (*p* < 0.001) and Fis1 (*p* < 0.01) (Figure 5A,B,E), indicating that diabetes not only decreases mitochondrial fusion but also decreases mitochondrial fission. It has been speculated that high-fat feeding and diabetes cause different degrees of damage to the mitochondrial dynamics. In addition, impaired mitochondrial dynamics may be tissue-specific.

Both morning and night exercises ameliorated diabetes-induced destruction of the mitochondrial networks (Figure 4A,B), abnormal mitochondrial morphology-lipid content, and swollen outer compartment in the mitochondria (Figure 4C). However, compared with morning exercise, night exercise had a more significant effect on improving the mitochondrial networks (Figure 4A,B) and morphology (Figure 4C). However, consistent with the expression of BMAL1, neither morning exercise or night exercise increased the expression of the molecules of the mitochondrial dynamics compared to DB mice (Figure 5A–E), which is also similar to our previous research [16]. The mitochondrial dynamics are closely related to mitophagy, and we found that diabetes significantly increased the mitophagy (Figure 5F–H). The function of Fis1 is much greater than that of mitochondrial fission [26,27]. Fis1 is involved in maintaining the normal mitophagy in mitochondria-rich slow muscle during exercise, which was reported in our earlier study. We recently reported that loss of Fis1 results in abnormal mitochondrial morphology and delayed mitophagy execution in skeletal muscle at rest and under exercise stress, indicating that Fis1 not only mediates fission but also maintains normal mitophagy [26,27,28]. Here, we found that diabetes downregulated the expression of Fis1 and also significantly upregulated the signal of mitophagy, probably because the decline of Fis1 expression further hindered the normal process of mitophagy.

Currently, numerous of reports have focused on the regulation of BMAL1 on mitochondrial dynamics, while there are fewer reports on the regulation of CLOCK on mitochondrial dynamics. Chip-sequencing of BMAL1 showed that regulators of mitochondrial fission, such as Fis1, and mediators of mitophagy, such as Pink1 and Bnip3, are both directly regulated by BMAL1. The expression levels of these molecules have also changed in mouse models that specifically knock out BMAL1 in the liver [29,30]. We found that T2DM upregulated the expression of CLOCK (Figure 3A,B) and downregulated the expression of OPA1 (Figure 5A,B) and Fis1 (Figure 5A,E) in the liver, and the expression of OPA1 and Fis1 were negatively correlated with the expression of CLOCK (Figure 6), suggesting that the regulation of the mitochondrial dynamics by CLOCK and BMAL1 may be opposite in the pathogenesis of T2DM. Knocking out liver BMAL1 disrupts the circadian rhythm of Bnip3 and reduces the level of mitophagy markers [31]. Here, we found that, while type 2 diabetes increased the expression of CLOCK (Figure 3A,B), diabetes also significantly increased the level of mitophagy (Parkin/LC3) (Figure 5F–H), suggesting that Pink1–Parkin and Bnip3 are different mitophagy pathways, and the regulation of CLOCK and BMAL1 on different mitophagy pathways may be specific.

Mammals have seven sirtuins (SIRT1–7), which are found in different subcellular locations [32]. CLOCK is, by itself, an acetyl transferase enzyme [33], which hints at the possibility that CLOCK and sirtuins could counteract each other on common targets. SIRT3, a mitochondrial sirtuin [34], mediates inner mitochondrial membrane fusion by deacetylating OPA1 [35]. Under stress conditions, OPA1 is hyperacetylated in mice and leads to mitochondrial fission [35]. The deacetylation of OPA1 by SIRT3 restores its activity [35], preserving the mitochondrial network and protecting cells from apoptosis [35]. These findings tie together CLOCK, sirtuins, and mitochondrial dynamics. Our results showed that diabetes upregulated the expression of CLOCK (*p* < 0.01; Figure 3A,B) and apoptosis (*p* < 0.05; Figure 7) and downregulated the expression of OPA1 (*p* < 0.001, Figure 5A,B), and the expression of CLOCK was negative correlated with the expression of OPA1, suggesting that SIRT3 may be involved in the regulation between CLOCK and OPA1. In addition, SIRT6 has also been demonstrated to regulate the hepatic circadian clock by controlling the recruitment to chromatin of CLOCK:BMAL1, and this results in the cyclic regulation of hepatic metabolism related to fatty acid and cholesterol metabolism [36], which hints at the possibility that SIRT6 is also involved in CLOCK–OPA1/Fis1. Since the close link between sirtuins and NAD^+^ has been extensively elaborated, CLOCK-driven NAD^+^ biosynthesis allows sustained sirtuin activity, impacting on nutrient handling and mitochondrial activity [9].

Mitochondrial dysfunction can induce apoptosis in several ways. Similar to the results of mitophagy in our study, diabetes significantly increased apoptosis (Figure 7A,B). Cytochrome C exists in the mitochondrial cristae in the form of a water-soluble protein [37]. Once stress occurs, cytochrome C is released from the mitochondria into the cytoplasm, resulting in apoptosis. In addition, OPA1 not only mediates mitochondrial fusion but also acts as a mitochondrial “shaping protein” that stabilizes the mitochondrial cristae, thereby preventing the release of cytochrome C and preventing apoptosis. Our results showed that T2DM downregulated OPA1 expression and upregulated apoptosis, suggesting that cytochrome C might be involved. Exercise at different times decreased apoptosis caused by diabetes, and night exercise was more advantageous than morning exercise in downregulating apoptosis signals (Figure 7A,B).

In conclusion, we demonstrated that both morning and night exercises ameliorated the increase in the blood glucose and T-CHO levels in the serum, improved mitophagy regulation, and reduced apoptosis caused by diabetes. However, morning exercise is superior to night exercise in enhancing insulin sensitivity and glucose transport, but night exercise is likely to improve mitochondrial networks and morphology through CLOCK–mitophagy and further alleviate apoptosis, thereby reducing fat infiltration and hyperlipidemia. In addition, we collected all of the liver tissue at around 8:00 a.m. on the second day after the exercise intervention. Given that type 2 diabetes can entrain the rhythmic fluctuations of the molecular clock, the expression of clock genes at different times is necessary to be determined in the future. Further investigations are also necessary to elucidate the precise relationship between circadian rhythm molecules and mitochondrial quality in chrono-exercise-regulated T2DM. CLOCK–OPA1/Fis1–mitophagy may be a novel and important target for diabetes therapy.

## 4. Materials and Methods

The Committee for the Care of Laboratory Animal Resources, East China Normal University approved all experimental protocols, and the study was conducted in accordance with the National Institutes of Health Guide for the Care and Use of Laboratory Animals (NIH Publications No. 8023, revised 1978).

### 4.1. Animals

Thirty 8–10-week-old male DB/DB (BSK.Cg-Dock7m +/+ Leprdb/JNju) mice, and ten 8–10-week-old male m/m (C57BLKS/JNju) mice were provided by the Model Animal Research Center of Nanjing University. The mice were housed in a hygienic animal facility at 22 ± 2 °C and 40–70% relative humidity with a 12/12 light–dark cycle (7:00 a.m.–7:00 p.m. was the light phase, and 7:00 p.m.–7:00 a.m. was the dark phase). The mice were randomly assigned to the control (WT), diabetes (DB), diabetic morning exercised (DM), and diabetic evening exercised (DE) groups (*n* = 10 each). The body weight and fasting blood glucose levels of the mice were measured during the experiment. All animal experiments were performed according to procedures approved by the Animal Experiment Committee of East China Normal University (m20200311).

### 4.2. Exercise

Mice from the DM and DE groups were acclimatized to the exercise environment by forcing them to run on the treadmill at a speed of 8 m/min, with an inclination of 0° for 10 min for 3 days. The mice were then forced to run on a treadmill at 13.3 m/min for an hour, with an inclination of 0° for five days per week for 8 weeks. Mice from the DM and DE groups ran at 8:00 a.m. and 8:00 p.m., respectively. Given mice are nocturnal animal and the light–dark cycle design as described in the Animals section, the morning exercise and night exercise in this study corresponded to the sleeping period and the active period of mice, respectively.

### 4.3. Blood Glucose Evaluation and Intraperitoneal Insulin Tolerance Test (ITT)

Mice were fasted overnight (approximately 16 h) every week prior to blood glucose tests. Blood samples were obtained from the tail vein, and blood glucose was measured using an ACCU-CHEK Active glucometer (Roche Holdings AG, Basel, Switzerland) with glucose test strips (Roche Holdings AG).

Insulin tolerance test (ITT): after a 4-h fast, mice (*n* = 4) were intraperitoneally injected with an insulin solution diluted in 0.9% saline (2.0 U/kg intraperitoneal injection, Sigma-Aldrich, St. Louis, MO, USA). Blood glucose levels were determined from saphenous vein blood 0, 30, 60, 90, and 120 min after insulin injection. The area under the curve (AUC) was determined using GraphPad Prism software, as described previously [38].

### 4.4. Serum Cholesterol (T-CHO) and Triglyceride (TG) Test

Blood was collected from the retro-orbital sinus of mice anesthetized with isoflurane using capillary tubes after the 8-week exercise (*n* = 6). Serum T-CHO (A111-1-1) and TG (A110-1-1) levels were examined using Nanjing Jiancheng kits. All tests were performed in accordance with the manufacturer’s instructions.

### 4.5. Immunohistochemical Staining

After harvesting the tissues of mice (*n* = 3), the livers were extracted and immersed in 10% neutral-buffered formalin overnight and dehydrated using 70% ethanol. Liver tissue blocks were embedded in paraffin, cut into 5-μm-thick sections, and transferred to slides. Tissue sections were stained with hematoxylin and eosin (Solarbio, Beijing, China, G1120). Five-micrometer-thick liver sections were incubated with primary antibody (GLUT4: Service-bio, Wuhan, China, GB11244; HSP60: Service-bio, Wuhan, China, GB11243) at 4 °C overnight, followed by incubation with a secondary antibody (Service-bio, GB23303) at room temperature for 1 h. The stained tissue sections were visualized and images were captured using an optical microscope (Olympus Optical Co., Ltd., Tokyo, Japan) and processed using Photoshop CS2 (brightness and contrast adjustments; Adobe, St. Jose, CA, USA). The positive area per unit area of tissue was quantified as described [39].

### 4.6. Preparation for EM

Targeted fresh tissues were selected to minimize the mechanical damage, then cut with a sharp blade and harvested quickly within 1–3 min. The 1-mm^3^ tissue blocks were transferred into an EP tube with fresh TEM fixative for further fixation at 4 °C for preservation and transportation. The tissues were washed using 0.1 M PB (pH 7.4) three times for 15 min. Tissues were further fixed with 1% OsO4 in 0.1 M PB (pH 7.4) for 2 h at room temperature, followed by dehydration at room temperature. The resin blocks were cut into 60–80-nm sections on an ultra microtome, and the tissues were fished onto 150 mesh cuprum grids with a formvar film. Images were captured using a transmission electron microscope (HITACHI; HT7800/HT7700). Image J was used on transmission electron microscopic images to observe individual mitochondrial morphology, as described previously [27].

### 4.7. Wes^TM^ Automatic Protein Expression Analysis

Liver tissues were collected at around 8:00 a.m. on the second day after the exercise intervention and put into liquid nitrogen for quick freezing. The liver tissues (40 mg for each sample, *n* = 3 to 4) were lysed in radioimmunoprecipitation assay (RIPA) buffer (0.1% sodium deoxycholate, 0.5% NP-40, 150 mM NaCl, and 50 mM Tris-Cl, pH 7.5). The protein concentration of the total homogenate was determined using the Bradford method (BCA Protein Assay Kit, 23227, Thermo Scientific^TM^, Waltham, MA, USA). The concentrations of all samples were diluted to 4.5 µg/µL with the sample buffer of the Wes^TM^ kit. Approximately 4.5 µL of the protein sample with 5 X Master Mix and diluted antibodies were added to each well to perform a Wes^TM^ automatic protein expression analysis (Revision 3.5, December 2018, Proteinsimple Co., Ltd., Silicon Valley, CA, USA. Proteins were probed using the high-sensitivity mix buffer (200 µL Lumino-S and 200 µL peroxide) and quantified using a Wes^TM^ automatic protein expression quantitative analysis system (Revision 3.5, December 2018, Proteinsimple (Shanghai) Co., Ltd., Shanghai, China).

### 4.8. Immunofluorescence

Five-micrometer-thick liver sections were removed from the obvious liquid, and the objective tissue was marked with a liquid blocker pen. Proteinase K working solution was used to cover the objectives and incubated at 37 °C for 25 min, followed by washing three times with PBS (pH 7.4) in a Rocker device for 5 min each (method for configuring a working solution of proteinase K, stock solution: PBS = 1:9). Sections were incubated with primary antibody (Parkin: Service-bio, 14060-1-AP; LC3: Service-bio, GB13431) at 4 °C overnight, followed by incubation with a secondary antibody coupled with Alexa fluor^®^ 594 (Thermo Fisher, A32740, 1:200 dilution) for 30 min at room temperature. Negative control sections were incubated with PBS (G4202-500 mL) instead of the primary antibody, and the rest of the steps were the same as those of conventional immunofluorescence. Nuclei were stained blue with DAPI. The TUNEL assay kit was purchased from Roche (Basel, Switzerland) and labeled with FITC. Cells that were positive for apoptosis were green. Images were captured using a Nikon Ortho-Fluorescent Microscope (NIKON ECLIPSE C1) equipped with a Nikon Imaging system (NIKON DS-U3) and processed using Photoshop CS2 (brightness and contrast adjustments; Adobe). The positive aera or positive puncta of per unit area of tissue are quantified as described [40].

### 4.9. Antibodies for Wes^TM^ Automatic Protein Expression Analysis

The following rabbit primary antibodies were diluted for immunoblotting: monoclonal antibody to GLUT4 (2213S, CST, 1:50), CLOCK (5157, CST, 1:50) and polyclonal antibody to BMAL1 (ARNTL) (14268-1-AP, Proteintech, 1:100). Polyclonal antibodies against OPA1 (sc-367890, Santa Cruz, 1:50), Mfn1 (ab57602, Abcam, 1:250), Drp1 (ab184248, Abcam, 1:250), Fis1 (10956-1-AP, Proteintech, 1:85), HSP90 (ab178854, Abcam, 1:250), β-Tubulin (AF7011, Proteintech, 1:50), and horseradish peroxidase-conjugated secondary antibodies were diluted 1:25 (Santa Cruz Biotechnology Inc., Dallas, TX, USA).

### 4.10. Statistical Analysis

Statistical analysis was performed as described previously [40]. Multiple comparison data were analyzed by one-way analysis of variance (ANOVA), and correlations were determined by Pearson correlations, both using GraphPad Prism software (version 5.0). Data are presented as the mean ± SD. *p*-values are shown as * *p* < 0.05, ** *p* < 0.01, *** *p* < 0.001, and **** *p* < 0.0001, unless indicated otherwise.

## Data Availability

The data that support the findings of this study are available from the corresponding author upon reasonable request.

## Supplementary Materials

The following supporting information can be downloaded at https://www.mdpi.com/article/10.3390/ijms23169308/s1.
