# Supplementary figures and images for: Chrono-Aerobic Exercise Optimizes Metabolic State in DB/DB Mice through CLOCK–Mitophagy–Apoptosis
Source: Int J Mol Sci. 2022 Aug 18;23(16):9308. doi: 10.3390/ijms23169308 (PMC9408978; doi:10.3390/ijms23169308)

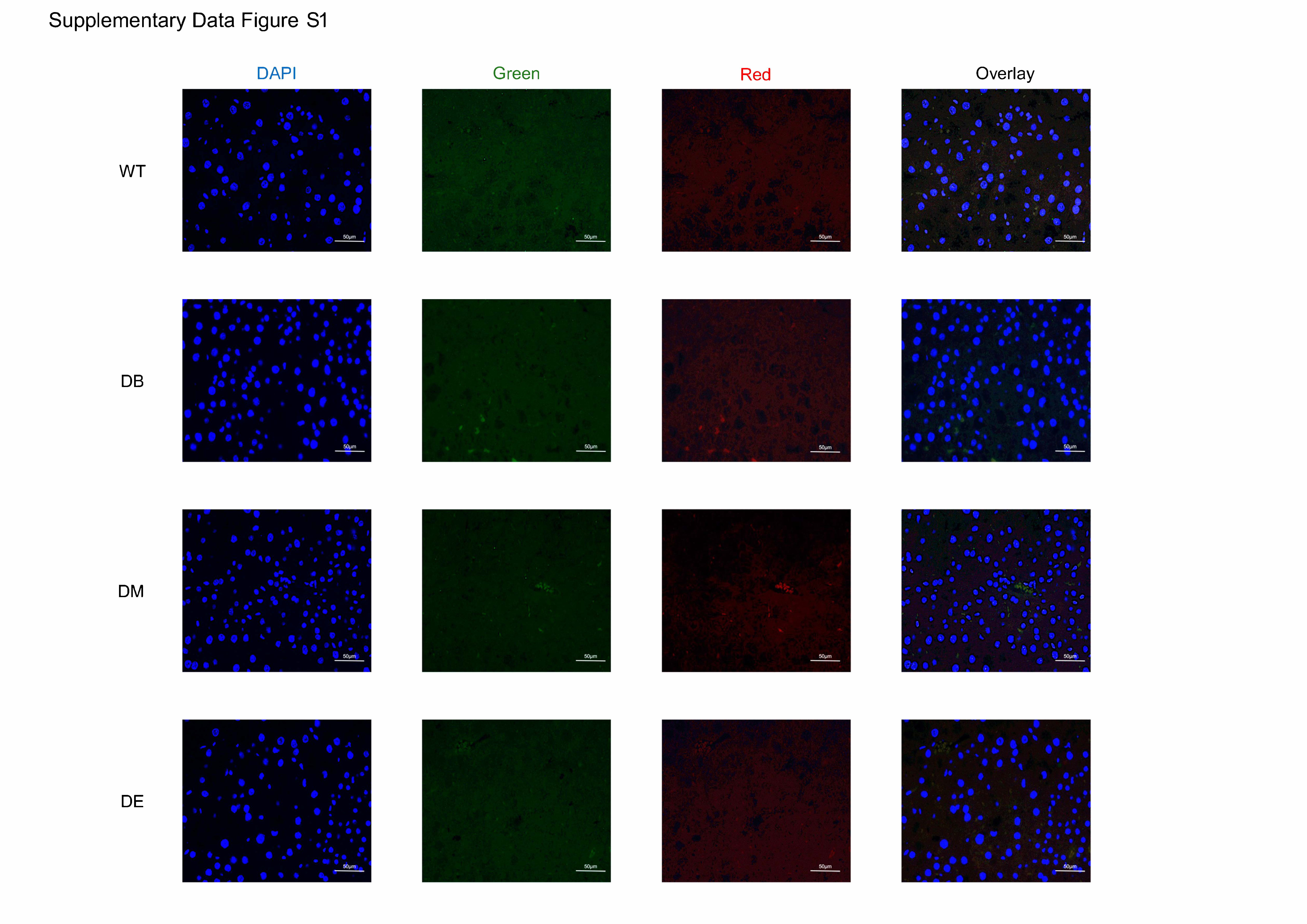

Supplement: Supplementary file 1 [file ijms-23-09308-s001.zip › ijms-1723683-supplementary.tif]
